# A rare cause of air-leak syndrome in a premature newborn: a case report

**DOI:** 10.1186/s12887-023-04140-9

**Published:** 2023-07-13

**Authors:** Ozan Uzunhan, Mihriban Begüm Yıldırım, Can Eser, Kazım Öztarhan, Alaettin Çelik

**Affiliations:** 1 Department of Neonatology, Demiroğlu Bilim University School of Medicine, İstanbul, Turkey; 2 Department of Pediatrics, Demiroğlu Bilim University School of Medicine, İstanbul, Turkey; 3 Department of Pediatric Cardiology, Demiroğlu Bilim University School of Medicine, İstanbul, Turkey; 4 Department of Pediatric Surgery, Demiroğlu Bilim University School of Medicine, İstanbul, Turkey

**Keywords:** Air-leak syndrome, Bronchial rupture, Persistent pneumothorax, Iatrogenic airway damage

## Abstract

**Background:**

Pulmonary complications, such as airway leak syndrome, are common in preterm neonates; however, bronchial rupture is a rarely seen phenomenon.

**Case Presentation:**

In this case, we present a preterm newborn who developed pneumomediastinum and pneumothorax. The pneumothorax persisted, despite placement of a thorax tube, requiring a thoracotomy to detect and treat the bronchial rupture.

**Conclusion:**

Physicians should have a high suspicion of bronchial rupture in patients with persistent air leak syndrome, even after thorax tube placement and continuous negative pressure implementation.

## Background

Over 3 million neonates die annually due to prematurity, making preterm birth the most common cause of neonatal mortality. Although the survival of preterm babies has increased dramatically over the last two decades, the immaturity of multiple organ systems leads to their increased susceptibility to complications and extended hospital stays [1]. Preterms at the lowest gestational age are at greatest risk for morbidity, with 93% having respiratory distress syndrome(RDS), 46% patent ductus arteriosus, 36% late-onset sepsis, 16% severe intraventricular hemorrhage, and 11% necrotizing enterocolitis [2].

The incidence of spontaneous pneumothorax is 1 to 2% of all live births, which is higher in preterm infants due to their anatomical differences [3–5]. Pneumothorax and pneumomediastinum in premature infants may result from tissue immaturity, mechanical ventilation, barotrauma, meconium aspiration syndrome (MAS), pneumonia and other infectious diseases, and iatrogenic causes [3]. The reported incidence of iatrogenic tracheobronchial injuries is approximately 0.005%, mainly resulting from single-lumen tube intubation, double-lumen tube intubation, and post-surgical tracheostomy [6]. Although regular suctioning is associated with shortened hospital stays and better outcomes, there are few case reports of tracheobronchial injury associated with endotracheal aspiration and one case report associated with nasopharyngeal suctioning [7–10]. In our case, we present a preterm intubated due to RDS who later presented with pneumothorax. Although a thorax tube was placed, the pneumothorax persisted, requiring a thoracotomy to detect and treat the bronchial rupture.

## Case presentation

A 27-week four days old preterm male infant was born from a dichorionic diamniotic twin pregnancy with low birth weight(1135 gr). His prenatal examinations were completed regularly without reported complications. In the 27th week of pregnancy, the mother experienced premature membrane rupture, contractions, and bleeding. She was treated with antenatal steroids and cesarean delivery was planned due to an arrested birth. During birth, the neonate was hypotonic and apneic, requiring intubation one minute after birth due to respiratory failure with an uncuffed endotracheal tube. His APGAR scores were 4,6 and 7 in the 1st, 5th, and 10th seconds, respectively. He was transferred to the newborn intensive care unit(NICU) for further management. After a diagnosis of respiratory distress syndrome was made, treatment with endotracheal poractant alfa 200 mg/kg, parenteral ampicillin-gentamicin, and caffeine citrate was initiated. His ventilation settings were arranged to assist control and volume guaranteed mode with maximum inspiring pressure of 20 cm/H²O. His cranial ultrasound showed no abnormalities.

On hospital day 3, he was extubated to nasal intermittent positive pressure ventilation but required reintubation for recurrent respiratory failure. His antibiotics were escalated to vancomycin-meropenem due to clinical deterioration.

**Fig. 1:**
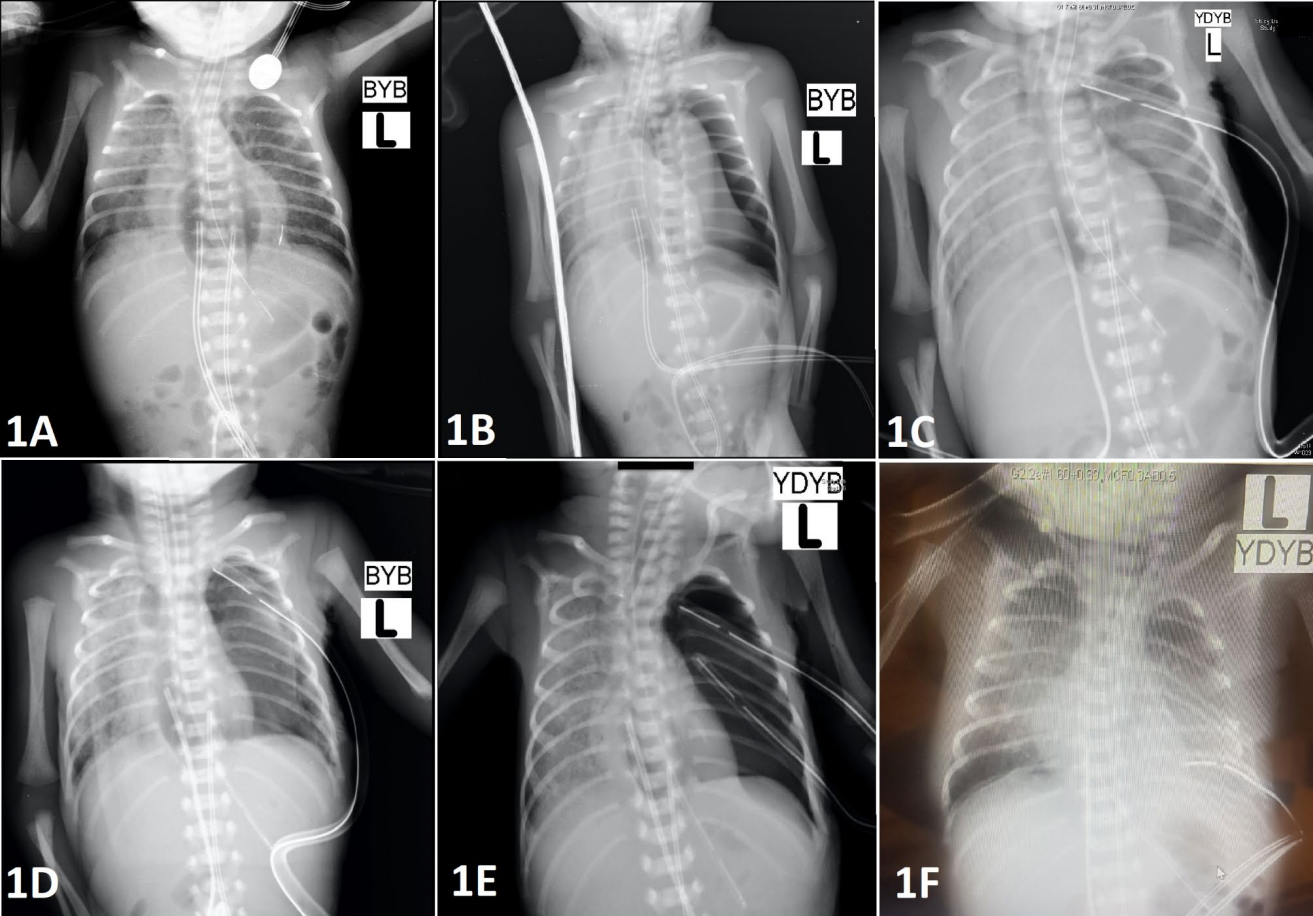
A: Initial X-ray showing pneumomediastinum. B: Left pneumothorax and mediastinal shift to the right side. C: Expanded left lung after thorax tube placement. D: Repeat pneumothorax in the left lung. E: Repeat pneumothorax, post-second thorax tube placement. F: After 1st thoracotomy, fully expanded left lung

During hospital day 5, the baby had worsening acute hypoxic respiratory failure. A chest x-ray revealed mediastinal pneumothorax (Fig. 1A), with a follow-up x-ray one hour later showing accelerated pneumothorax on the left side and mediastinal shift to the right.(Fig. 1B) To treat the pneumothorax, a thorax tube was placed through the left 5th intercostal space. Repeat x-rays showed an expanded lung on the left side and air bronchograms on the right(Fig. 1C). Ten hours later, he had persistent acidosis and hypoxia, with another x-ray displaying a repeat pneumothorax on the left side(Fig. 1D). The thorax tube was set to continuous suction, resulting in a reduction of pneumothorax on subsequent imaging.

On the 6th day of hospitalization, an echocardiogram revealed a 3 mm patent ductus arteriosus(PDA) with mostly left to right two-way shunts and pulmonary hypertension. His oxygen saturation levels were approximately 80%, and his arm-leg saturation differences were higher than 15 mm Hg, suggesting differential cyanosis. These findings were suspicious for persistent fetal circulation; therefore, pediatric cardiology advised against the closure of PDA. A new chest x-ray showed a collapsed left lung(Fig. 1E), and a second chest tube was placed in the left hemithorax.

The decision to perform a surgical intervention was made given no resolution of the pneumothorax after the second chest tube and the patient’s continued clinical deterioration. A left posterolateral thoracotomy was performed. During the exploration, a 1 cm perforated area between the left bronchus and carina was found by following the air leak through the visceral pleura. The perforated area was repaired by using a pleural patch, and suturing with 6.0 prolene was performed. At the end of the procedure, there was no visible bleeding or air leak. The anesthesiologist applied high-pressure air via the endotracheal tube to check for any air leaks. A chest tube was inserted, and the thoracotomy incision was closed by layers. Post-procedure chest x-ray showed an expanded left lung. (Fig. 1F)

**Fig. 2:**
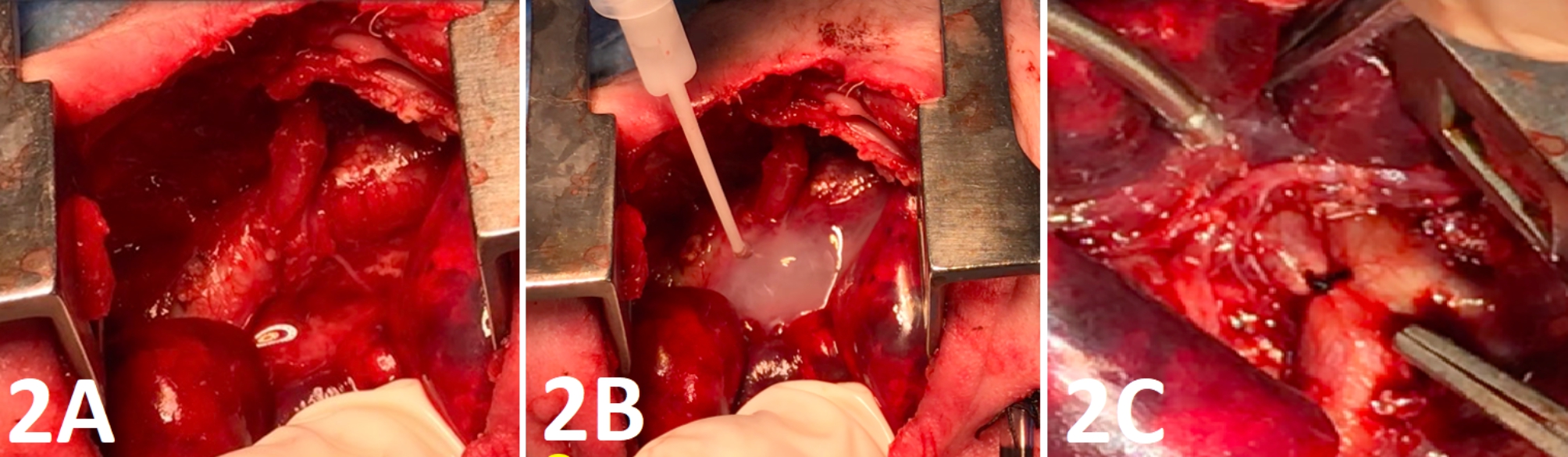
A: The perforated area was repaired by using a pleural patch, and the left 5th intercostal muscle long peduncle. B: Fibrin glue and spongestan were used as an anchorage on top of the patch. C: Large PDA was closed with one silk suture

Three days later, the neonate experienced another episode of hypoxia, which was found to be secondary to a repeat left-sided pneumothorax. Consequently, a second thoracotomy was performed using the same technique as the prior surgery, with an air leak emanating from the same area. The perforated area was repaired using a pleural patch and the left 5th intercostal muscle long peduncle (Fig. 2A). Fibrin glue and spongestan were used as anchorage on top of the patch (Fig. 2B). After the repair, no leak was found with the use of maximal airway pressure. A PDA ligation operation was also performed simultaneously during the procedure (Fig. 2C). There was no air leakage in the repeat x-ray. Both the first and second thorax tubes were removed on postoperative days 0 and 5, respectively. There were no signs of air buildup or pneumothorax on the following days. On the 161st day of admission, the patient was discharged home with a home-type ventilator and 30% oxygen support.

## Discussion

Airway leak syndrome occurs when air escapes from the lung into extra-alveolar spaces causing respiratory symptoms. A variety of clinical presentations may be seen depending on the location of the air, which can include pneumothorax, pneumomediastinum, pulmonary interstitial emphysema, and pneumopericardium. Known predisposing factors for airway leak syndrome are prematurity, RDS, and bronchitis [7]. Our case was a 27-week-old premature infant with RDS but had no signs of bronchitis like fever or leukocytosis.

Pulmonary barotrauma can be another cause of airway leak syndrome; though, large airways are less likely to be damaged by barotrauma, with injury more commonly affecting the distal airways. The pathophysiology of barotraumatic air leak typically begins with overdistended alveoli, leading to alveoli rupture, not bronchial rupture [11]. Among iatrogenic causes, the most common etiology of tracheobronchial injury is acquired damage during intubation [12]. In our case, this preterm infant was intubated immediately after birth, re-intubated on his 3rd day, and subsequently deteriorated on day 5. It is not suspected that injury occurred from intubation, as the deterioration started 2 days after re-intubation and, endotracheal tube was in the proper position on all imaging studies. There are previously reported pediatric cases of premature neonates who were intubated and experienced air leaks with endotracheal tube suctioning [8, 9]. The mechanism of injury in these reports were mainly from tissue trauma causing perforations in the trachea or the right bronchial tree. The respiratory system’s cartilage tissue development begins anywhere from the 51-54th day of gestational life. According to Carnegie staging, tracheal cartilage tissue development starts as a ventromedial mesenchymal condensation and continues throughout pregnancy until it reaches its mature characteristic horseshoe shape, which wraps around the trachea 320 degrees [13]. However, in preterm neonates, the immaturity of this cartilagenous tissue makes the trachea more susceptible to rupture. Eckenhoff et al. describes that, unlike adults, the cricoid ring is the narrowest part to encounter during any airway intervention until the age of 8 [14]. Additionally, the cricoid ring mucosa lacks submucosa, making this location particularly vulnerable to iatrogenic damage from an instrument. The anatomical position of the cricothyroid membrane is also forced into a cephalic shape, in contrast to a cylinder shape in adults. Due to this difference in position, if the cricothyroid membrane is iatrogenically penetrated, perforation is expected to lead into the glottic area, not the trachea [14].In our case, the location of the rupture was between the carina and the left bronchus, which is the cartilaginous anteroinferior part of the bronchus, drawing us away from the possibility of barotraumatic etiology. Although the exact cause of the large bronchial rupture remains unknown, we suspect the suction catheter used for endotracheal aspiration perforated the carina, given the fragility of the cartilaginous tissue, as described above.

## Conclusions

Physicians should maintain a high index of suspicion for bronchial rupture in patients who have persistent air leak syndrome after thorax tube placement and continuous negative pressure implementation. To prevent such iatrogenic bronchial ruptures, healthcare workers should be cautious during intubation and aspiration of premature newborns. Surgical treatments such as pleural and intercostal muscle patching, fibrin glue, and spongestan should be considered as possible treatment options.

## Data Availability

Not applicable.

## List of abbreviations

MAS: Meconium Aspiration Syndrome
NICU: Newborn Intensive Care Unit
PDA: Patent Ductus Arteriosus
RDS: Respiratory Distress Syndrome
